# Optimization of Proprioceptive Stimulation Frequency and Movement Range for fMRI

**DOI:** 10.3389/fnhum.2018.00477

**Published:** 2018-12-03

**Authors:** Timo Nurmi, Linda Henriksson, Harri Piitulainen

**Affiliations:** ^1^Department of Neuroscience and Biomedical Engineering (NBE), Aalto University, Espoo, Finland; ^2^Aalto NeuroImaging, Aalto University, Espoo, Finland

**Keywords:** kinesthesia, passive movement, proprioception, somatosensory cortex, repetition rate, sensorimotor system, fMRI, movement range

## Abstract

For vision, audition and tactile sense, the optimal stimulus frequency for fMRI is somewhat known. For proprioception, i.e., the “movement sense”, however, the optimal frequency is unknown. We studied the effect of passive-finger-movement frequency on proprioceptive fMRI responses using a novel pneumatic-movement actuator. Eleven healthy right-handed volunteers participated in the study. The movement actuator passively moved the participant’s right index finger at frequencies of 0.3, 1, 3, 6, 9, or 12 Hz in a blocked design. A functional localizer was used to define regions-of-interest in SI and SII cortices. In addition, effect of movement range on the fMRI responses was tested in a separate session with 1, 3, 5, and 7 mm movement ranges at a fixed 2 Hz frequency. In primary somatosensory (SI) cortex, the responses were stronger at 3 Hz than at 0.3 Hz (*p* < 0.001) or 1 Hz (*p* < 0.05), and at ≥6 Hz than 0.3 Hz (*p* < 0.001 for frequencies ≥ 6 Hz). In secondary somatosensory (SII) cortex, all movements, except at 0.3 Hz, elicited significant responses of similar strength. In addition, 6, 9, and 12-Hz movements elicited a significant offset response in both SI and SII cortices (*p* < 0.001–0.05). SI cortex required a total stimulation duration of 4 min to elicit significant activations at the group-level whereas for SII cortex 1 min 20 s was sufficient. Increase in the movement range led to stronger responses in SI cortex, but not in SII cortex. Movements above 3 Hz elicited the strongest SI cortex responses, and increase in the movement range enhanced the response strength. We thus recommend that movements at *3–6 Hz* with a movement range of 5 mm or higher to be used in future studies of proprioception. Our results are in-line with previous fMRI and PET studies using tactile or median nerve stimulation at different stimulation frequencies.

## Introduction

The term proprioception, i.e., the position and movement sense of the body, was first introduced by Sherrington (1907), who described proprioceptors as: “In muscular receptivity we see the body itself acting as a stimulus to its own receptors—the proprioceptors” (for a review, see Proske and Gandevia, 2012). Proprioceptors are located in muscles and joints and are thus sensitive to changes in the internal state of the musculoskeletal system. For example, muscle spindles are sensitive to changes in the length and stretch of the muscle, and GTOs are monitoring tension produced by the muscle (Moore, 1984; for a review, see Proske and Gandevia, 2012). Proprioceptors include also joint receptors and even some cutaneous receptors (Ferrell et al., 1987). Proprioception is crucial for continuous, smooth motor actions (Forget and Lamarre, 1987; Ghez et al., 1995; Messier et al., 2003). Thus, it is not surprising that many motor disorders, such as cerebral palsy and Parkinson’s disease, are accompanied with deficits in proprioception (Zia et al., 2000; for a review see Konczak et al., 2009). Despite its relevance to motor control and motor disorders, proprioceptive processing in the human brain is still inadequately understood.

Consequently, the optimal parameters for proprioceptive stimulation to elicit the maximal hemodynamic fMRI responses are unknown. Selecting frequency of sensory stimuli that maximizes the brain activation in fMRI and minimizes scanning time enables efficient experimental setup. In the visual domain, strongest responses are obtained by a flickering 8–15 Hz stimulus in primary visual cortex for both PET (Fox and Raichle, 1984) and fMRI (Kwong et al., 1992).

Optimal stimulation frequency might also depend on the brain region in question. For example, the optimal parameters differ between the visual areas (Singh et al., 2000; Henriksson et al., 2008). The same phenomenon has been observed for auditory stimuli. Response in lower-level regions of the auditory cortex, such as the inferior colliculus, increase as with stimulation frequency, whereas the higher-level regions, such as medial geniculate body and superior temporal gyrus, initially increase with stimulation frequency from 2 to 10 Hz (Heschl’s gyrus) or from 1 to 2 Hz (Superior temporal gyrus) but then begin to decrease with further increase in the stimulation frequency from 10 to 35 Hz (Heschl’s gyrus) or from 2 to 35 Hz (Superior temporal gyrus; Harms and Melcher, 2002).

Optimal stimulation frequency for somatosensory senses has been studied in fMRI using tactile stimulation and electric median nerve stimulation. When using tactile stimulation, the Brodmann area 3b in SI cortex was deemed to be sensitive to frequency with the response getting stronger as the stimulation frequency increased from 1 to 4 Hz, but plateaued with further increase to 10 Hz (Hlushchuk et al., 2015). With rats, similar results were obtained with tactile stimulation of the forepaw, where frequencies of 1.5–3 Hz elicited the strongest responses in fMRI (Sanganahalli et al., 2008). In the case of median nerve stimulation, increasing the stimulation frequency from 0.2 or 0.5 Hz to 3 or 5 Hz significantly increases the response strength in SI cortex when using PET (Ibáñez et al., 1995) or fMRI (Iramina et al., 2001; Ferretti et al., 2005). Further increases in the frequency have been ineffective (Manganotti et al., 2009). Conversely, SII cortex seems to be invariant for changes in frequency or amplitude of the median nerve stimulation (Backes et al., 2000; Ferretti et al., 2005). Even though median nerve stimulation likely activates a mixture of tactile and proprioceptive afferents, it cannot be reliably deduced that 3–5 Hz is the optimal stimulation frequency also for the proprioceptors in fMRI.

Proprioceptors have been stimulated in fMRI by using a MRI-compatible pneumatic vibration on skin over a tendon. It has been well established that ∼80 Hz vibration efficiently activates muscle spindles and produces a proprioceptive illusion of movement (Goodwin et al., 1972; Roll and Vedel, 1982). Similar high-rate vibration activates SI cortex when measured using fMRI (Montant et al., 2009; Goble et al., 2011). Proprioceptors can be stimulated at more “natural” rates by passively moving the participant’s limbs. In fMRI studies, most often the passive movements have been generated manually by the investigator (Carel et al., 2000; Guzzetta et al., 2007; Boscolo Galazzo et al., 2014). The drawbacks of experimenter-evoked movements are variable stimulus frequency and amplitude. A more stable approach is to use movement actuators that passively move the participant’s limb. Previous studies have used MRI-compatible device, capable to move index finger along one axis (Van de Winckel et al., 2013a) or two axes (i.e., both vertically and horizontally). The two-dimensional passive movements allows discrimination tasks of shapes such as rectangles and triangles, which have been investigated in healthy participants, patients with stroke, and children with cerebral palsy (Van de Winckel et al., 2005, 2012, 2013b). However, until now, only fixed movement frequency has been applied in fMRI-studies (Weiller et al., 1996; Yu et al., 2008, 2011; Van de Winckel et al., 2013a; Tang et al., 2015). In addition to functional studies, a robotic device has also been used to asses proprioceptive capability which was then correlated to diffusion tensor imaging (DTI) results in children with perinatal stroke and cerebral palsy (see Kuczynski et al., 2017, 2018). Moreover, motor system decline and proprioception has been studied with resting state fMRI (Mathys et al., 2014). In MEG which measures the neuronal activity more directly (Hämäläinen et al., 1993), the optimal ISI in eliciting the strongest proprioceptive responses in a given time appears to lay within 1.5–3.0 s for index-finger movements (Smeds et al., 2017). However, clear MEG responses are detected even at 12-Hz movements (Piitulainen et al., 2015).

**FIGURE 1:**
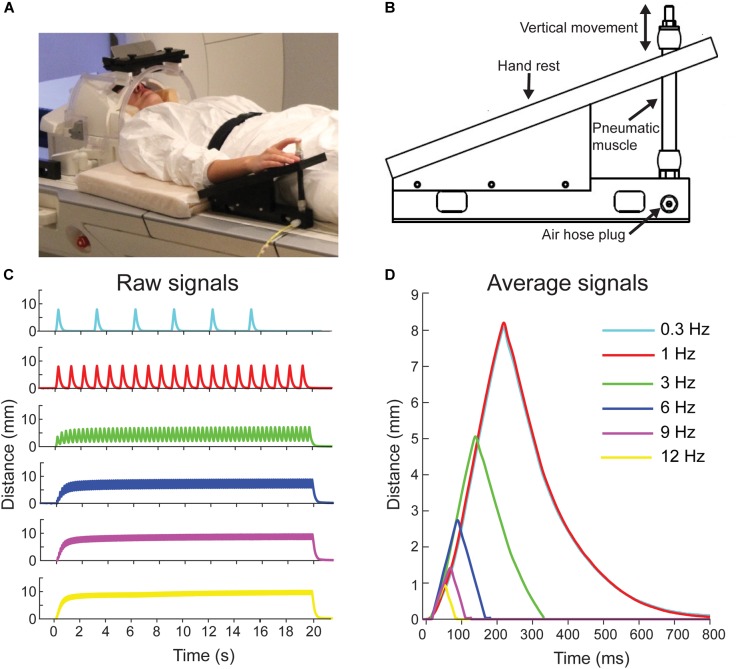
Pneumatic movement actuator and movement properties. **(A)** Participant is lying on the fMRI with her right index finger attached to the movement actuator. Written consent was obtained from the participant for the photograph to be used in a publication. **(B)** Schematic of the pneumatic-movement actuator. **(C)** Finger position for a one 20-s stimulation block at the different movement frequencies. **(D)** Average finger positions for the movement frequencies across three blocks. Color codes are the same in all panels.

The primary aim of this study was to determine the optimal finger-movement frequency for proprioceptive stimulation, i.e., to elicit the maximal hemodynamic fMRI responses in SI and SII cortices. Based on previous findings on somatosensory stimulation in fMRI, our hypothesis was that movements at 3–5 Hz would be the most effective to elicit SI cortex responses, and that SII cortex would be less sensitive to the stimulation frequency (Iramina et al., 2001; Ferretti et al., 2005; Hlushchuk et al., 2015). We also examined the temporal dynamics of the hemodynamic responses in SI and SII cortices. In addition, we aimed to pinpoint the main cortical response locations for the proprioceptive stimulation of the right-index finger, and determined the sufficient stimulation duration for pinpointing the response locations. Finally, because we varied the movement frequency while keeping the velocity of the stimuli constant, the total movement range inevitably decreased with increasing stimulus frequency (see Figure 1D). Therefore, we performed a control experiment to examine whether the movement range affects the fMRI response strength by varying the movement range at a fixed 2 Hz-movement frequency.

## Materials and Methods

### Participants

Eleven healthy right-handed volunteers (three women, age: mean ± standard deviation, range, 30.7 ± 7.1 years, 22–41 years) participated in the study. All volunteers participated both the main experiment and the control experiment. Edinburgh Handedness Inventory was used to determine participants’ handedness (test scale: –100 [purely left-handed] –100 [purely right-handed]). Participants were right-handed (81.6 ± 19.7, range: 42–100). This study was carried out in accordance with the recommendations of the Declaration of Helsinki, and the ethics committee of Aalto University. The protocol was approved by the ethics committee of Aalto University. All subjects gave written informed consent in accordance with the Declaration of Helsinki.

### MRI Scanner and the Scanning Parameters

The structural and functional imaging were performed using a 3T MAGNETOM Skyra whole-body scanner (Siemens Healthcare, Erlangen, Germany). A 32-channel head coil was used. All measurements were done at the Advanced Magnetic Imaging (AMI) Centre of Aalto NeuroImaging, Aalto University, Espoo, Finland.

Anatomical T1-weighted MPRAGE images were obtained with 176 slices and a 256 × 256 grid with a 256 × 256 mm field-of-view (FOV). This yielded a voxel size of 1 × 1 × 1 mm. Orientation was sagittal. Repetition time (TR) was 2.53 s and echo time (TE) was 3.3 ms. Flip angle was 7°.

For all functional scans, a standard EPI sequence was used with a TR of 2.5 s and TE of 30 ms. Orientation of the images was axial oblique. A functional volume consisted of 44 slices with a 64 × 64 matrix in each slice with a FOV of 192 × 192 mm. This yielded a voxel size of 3 × 3 × 3 mm. Flip angle was 90°.

### Proprioceptive Stimulation

Figure 1A shows a participant with the novel in-house-built, fMRI-compatible pneumatic-movement actuator that evoked flexion-extension movements of the right index finger at frequencies between 0.3 and 12 Hz (see Experimental procedure). The movement actuator consisted of an elastic pneumatic artificial muscle and a supporting plastic frame (Figure 1B; for further details of operating principle see: Piitulainen et al., 2015). In brief, index finger was attached to a pneumatic artificial muscle (DMSP-10-100 AM-CM, Festo AG & Co., Esslingen, Germany) that moved downward in vertical direction when its internal air pressure was increased to 5 bar, thereby flexing the finger, and then returned back to the initial position when the air pressure was released (extension of the finger). Movement stimuli were controlled using Presentation Software (ver. 18.1, Neurobehavioral Systems, Albany, CA, United States).

The range of the vertical movements varied with movement frequency: 8.1 mm at 0.3 Hz, 8.2 mm at 1 Hz, 5.1 mm at 3 Hz, 2.7 mm at 6 Hz, 1.4 mm at 9 Hz, and 0.9 mm at 12 Hz. These ranges were measured using a laser device (HG-C1100, Panasonic, Japan). See Figures 1C,D for position signals of the evoked movements.

For the control experiment, stimulation frequency was kept constant at 2 Hz and the movement range was either 1, 3, 5, or 7 mm (four conditions).

### Experimental Procedure

During scanning, the participants were laying on the scanner table on their back. For comfort, a pillow was placed under the participant’s hamstrings and calf (Figure 1A). Participant’s right hand and distal part of the forearm rested on the upper plate of the movement actuator. The tip of the participant’s right index finger was taped to the head of the artificial muscle of the movement actuator using surgical tape. During fMRI scanning, a still image was presented to the participant using a projector (located outside the MRI room) with mirrors and a back-projection screen. The participant was instructed to relax and fixate at the image.

In the (f)MRI session, three functional task runs were first performed followed with an anatomical T1-weighted MPRAGE scan. The first task run lasted 25 min and 8 s. Then, a separate functional localizer run was presented for 5 min and 8 s, and was followed with the second task run. The task runs were identical in structure.

The task and localizer runs were block-design runs where a 20-s-stimulation block always alternated with a 20-s-rest block. Task runs consisted of six conditions differing in movement frequency (0.3, 1, 3, 6, 9, and 12 Hz). These frequencies were presented in a block list that included each movement frequency once and the rest block in between them. The order of frequencies was randomized within each block list. Block lists were repeated 12 times in total—in two task runs (6 block list in each). Thus, the total stimulation time was 4 min for each movement frequency.

The localizer run had similar block design and a block list structure as the task runs. The only differences were the length (5 min 8 s in total with rest blocks included, 2 min with stimulation blocks only) of the localizer run, and that within each task block, a random sequence of the movement frequencies was used. The stimulation frequencies were the same as in the task run but each movement frequency was repeated only for four cycles at a time.

The control experiment was recorded in a separate session and consisted of two runs with total 10 repetitions (5 repetitions per run) of each of the four different movement ranges (presented once within each stimulation block list). Otherwise, the experimental parameters were identical to those of the main experiment.

### Analysis of fMRI Data

Participant-wise preprocessing was done using a custom Matlab (R2016b, Mathworks, Natick, MA, United States) script that used SPM12 (Wellcome Department of Imaging Neuroscience, University College London, United Kingdom) functions.

First, the data were converted from Dicom to NIfTI format. For the group-level analysis, but not for the ROI-based analyses, the data were spatially normalized to a common MNI space. In order to be better align with the MNI space, the origin of the anatomical image was automatically set to the anterior commissure and anatomical image was moved and reoriented so that its anterior-posterior-commissure-line corresponded to the axial plane using spm_auto_reorient script (by Carlton Chu, UCL, London, United Kingdom and Christophe Phillips University of Liège, Belgium). The fMRI data were then slice-time-corrected, motion-corrected, realigned to the last functional volume, functional volumes co-registered to anatomical volume, and smoothed with a kernel of 6 mm using SPM functions. Next, all of the participants’ volumes were segmented and normalized to a common MNI template. A temporal high-pass filter with a 754 s cutoff was used for the task runs and a 154 s cutoff for the localizer run. The cutoff was defined to be half of the run duration.

Then, timing and order of the stimulus blocks were extracted, and a design matrix was constructed accordingly. The columns of each frequency in the design matrix were convolved with a standard hemodynamic response function (HRF). In addition, nuisance regressors that represented movement and rotation of participant’s head in three axes were included in the design matrix (movement and rotation regressors in *x, y*, and *z*-axes). General Linear Model (GLM) was used to obtain the beta weights for each frequency. Finally, SPM contrast images were constructed for each frequency using beta weights from the two task runs. Identical procedure was used to construct localizer and the control experiment contrast images for each participant for the single localizer and the four control experiment conditions respectively.

#### Regions of Interests and BOLD-Time-Courses

We used the functional localizer results to semi-manually define functional regions-of-interests (ROIs) using Marsbar (MARSeille Boîte À Région d’Intérêt; Marseille, France; version: 0.44) toolbox. The SI and SII cortices of the left hemisphere were selected as the ROIs. To define the ROIs a threshold of *p* = 0.001 (uncorrected) was used for contrast SPM images of the localizer scan in each individual separately. If needed, the initial threshold was then adjusted by comparing the extent of activation to anatomy to yield the final ROI cluster.

Next, the average time-courses of percent signal change in each ROI were computed using finite-impulse response (FIR) model from the Marsbar toolbox. Temporal resolution of the time-courses was 2.5 s (equal to one TR). The time courses were averaged across the voxels in each ROI and across the 12 blocks for each participant. Then the time-courses were averaged across the participants.

As a comparable metric to the real time-courses, a canonical haemodynamic response function (cHRF) was convolved with a 20-s-boxcar function to yield a theoretical response to the 20 s stimulation and shown against the time-courses.

The same ROIs from the main experiment were used for the control experiment. Since the control experiment was performed in a separate session, the anatomical and functional scans of the control experiment were co-registered to the anatomical image of the main experiment. The aforementioned steps matched the volumes of the control and main experiments to the same coordinate system.

### Statistical Analyses

#### Response Strengths

The response strength for the movement frequencies was defined as the mean beta-weight over the voxels of each of the ROIs. A non-parametric repeated measures Friedman’s test (Friedman, 1937) was used to denote possible statistically significant differences between the frequencies. Friedman’s test was used because the mean beta values were not normally distributed (Kolmogorov–Smirnov test: SI cortex: *p* < 0.05 for 0.3 Hz, *p* < 0.01 for all other betas, SII cortex: *p* < 0.05 for 0.3 and 12 Hz, *p* < 0.01 for all other betas) and sample size was relatively small. In case of significant differences, Conover *post hoc* test (Conover, 1999) was used to determine possible differences between the specific frequencies. Statistical analysis was done using R statistical software (version 3.3.1).

An identical analysis was performed for the control experiment data to obtain the response strengths for the four different movement ranges.

#### Response Locations

Response locations were defined at group level only. These group-level results were obtained by taking the individual-level contrast images and doing a random-effects group-level analysis (one-sample *T*-test) to them. Cluster-extent-based thresholding was used where, at first, an initial, voxel-wise threshold *p*-value of 0.001 (uncorrected) was used to obtain the activation clusters. Next, a cluster-level-false-discovery-rate (FDR) correction with a *p*-value threshold of 0.05 was applied to the clusters by selecting such a cluster-extent-threshold (*k_e_*) that only clusters with a *p*-value equal to or above the FDR threshold survived the thresholding. The group-level results are in MNI space. In addition, the mean center, mean distance from the mean center and standard distance were calculated for the ROIs.

#### Response Time-Courses

Using the R software, a non-parametric Friedman’s test was used (the distribution of percent signal change-values at a time point of the time-courses were non-normal) to denote significantly different amplitude of time points measured by percent signal change level of the frequency-dependent-time-courses. In case of significant difference, Conover’s *post hoc* test (FDR-corrected for multiple comparison) was used to indicate the frequencies that differed in the given time point.

#### The Effects of Stimulation Duration

The effect of the stimulation duration on the response strength was examined by repeating the analysis using 4, 6, 8, 10, or 12 block lists—i.e., different stimulation durations. For clarity, this analysis was limited to the 3-Hz frequency only. Analysis was performed identically as the analysis of the group-level response locations (see “Response locations” above). Finally, significant activation clusters were determined in the group-level for the tested stimulation durations with the same procedure and parameters as with determining the group-level response locations.

## Results

Our novel movement actuator (see Figure 1) did not cause visible interference to the magnetic field of the MRI scanner and proved as feasible tool to study proprioception using fMRI.

### Response Strength

**FIGURE 2:**
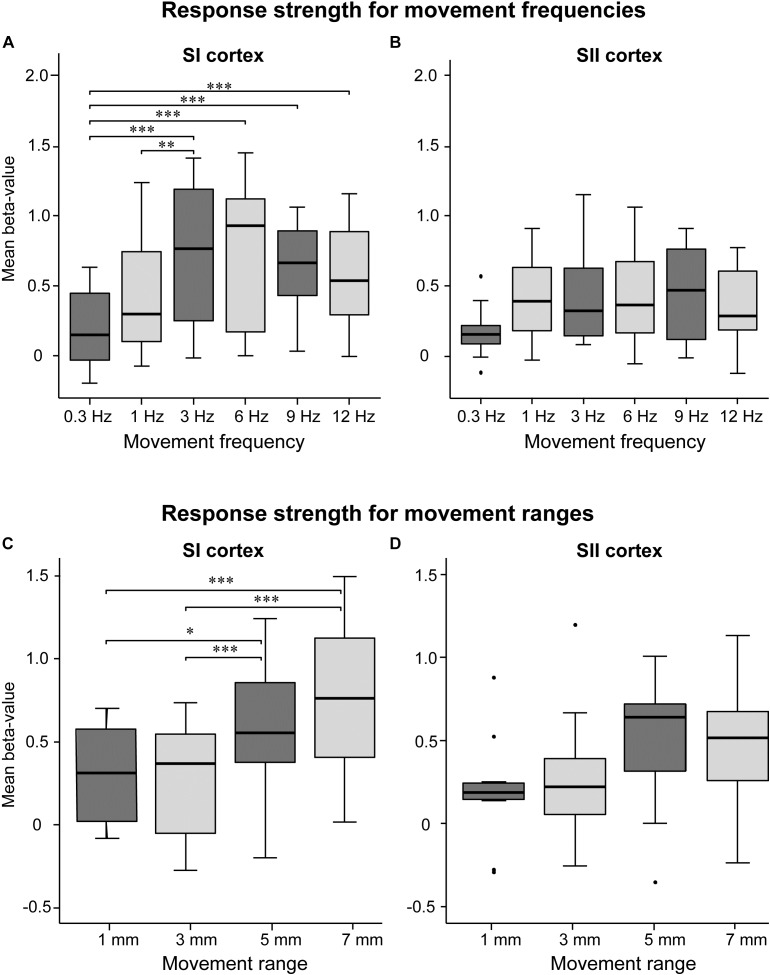
Effect of movement frequency and range. Mean beta-values in the SI **(A)** and SII **(B)** cortices for movement frequency (**A,B**, upper panels) and range (**C,D**, lower panels; *n* = 11). The black horizontal line inside the box represent median value. The horizontal boundaries of the boxes represent quartiles. The whiskers indicate the range, excluding outliers that are indicated with black dots. ^∗^*p* < 0.05, ^∗∗^*p* < 0.01, and ^∗∗∗^*p* < 0.001. Please note that the mean beta values between movement frequency and range are not commensurable since they were recorded in separate sessions.

Figure 2 shows response strengths (beta-weights) for SI or SII cortices. In SI cortex, the response strength differed between frequencies (*p* < 0.001, χ^2^= 21.03, DF = 5). The response at 3-Hz movement was significantly stronger compared to the movements at 0.3 Hz (*p* < 0.001) and 1 Hz (*p* < 0.05). However, the response strengths at 3, 6, 9, and 12 Hz were at the same level (*p* = 1.0 for all). The movements at the lowest 0.3 Hz frequency produced significantly weaker responses than all other tested frequencies (*p* < 0.001), except at 1 Hz (*p* = 0.14). In SII cortex, the response strength did not differ between the frequencies (*p* = 0.39, χ^2^ = 5.2, DF = 5; Figure 2B).

Figures 2C,D shows response strengths for the control experiment in which the movement range was varied while keeping the movement frequency constant at 2 Hz. In SI cortex, the response strength differed between the movement ranges (*p* < 0.01, χ^2^ = 15.98, DF = 3). The 7-mm movement elicited significantly stronger responses than 3-mm (*p* < 0.001) or 1-mm (*p* < 0.001) movements, but did not differ from the 5-mm movement (*p* = 0.150) In addition, the 5-mm movement elicited stronger activations than the 3-mm movement (*p* < 0.001) and the 1-mm movement (*p* < 0.05). In SII cortex, no significant main effect of the movement range was observed (*p* = 0.094, χ^2^ = 6.38, DF = 3).

### Response Location

**FIGURE 3:**
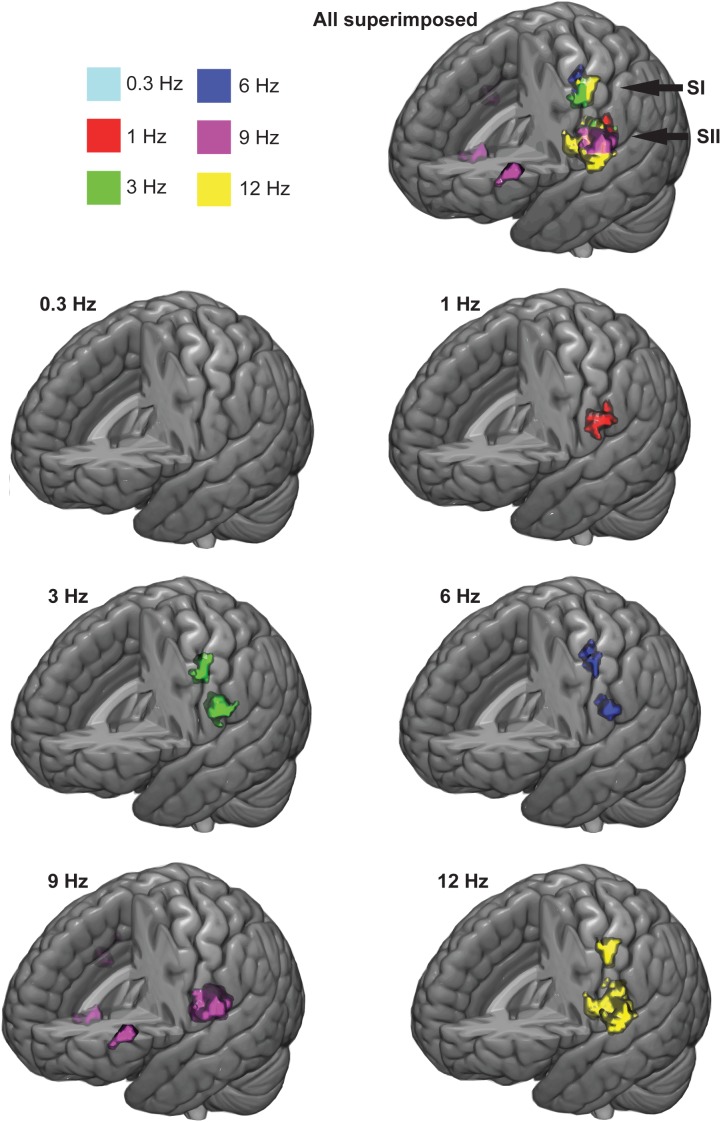
Group-averaged response clusters. The significant cortical clusters are colored for each movemet frequency separately. Cluster-wise threshold was 0.05 (FDR-corrected) and voxel-wise threshold was 0.001 (uncorrected).

Figure 3 shows the significant response clusters at the group level, and Table 1 presents the respective MNI coordinates from the main experiment. Most of the significant clusters were seen in SI and SII cortices in the contralateral hemisphere in relation to side of the stimulated finger.

**Table 1:** Centers of mass for group-average response clusters in SI and SII cortices (*x, y*, and *z* MNI-coordinates).

Movement	SI center of mass	SII center of mass
0.3 Hz	–	–
1 Hz	–	–46.0, –29.0, 20.3
3 Hz	–44.9, –16.7, 48	–43.8, –28.5, 19.8
6 Hz	–37.3, –21.8, 49.2	–41.8, –29.6, 19.1
9 Hz	–	–49.3, 25.2, 16.5
12 Hz	–47.1, –19,2, 49.6	–49.3, –21.2, 14.6

At the group level, movements at 3, 6, and 12 Hz elicited statistically significant response clusters in both SI and SII cortices in the left hemisphere (voxel-wise, initial threshold: *p* < 0.001, uncorrected; cluster-wise threshold *p* < 0.05, FDR-corrected). In contrast, movement at 0.3 Hz failed to elicit significant response clusters at the group level. The movement at 1 Hz yielded a statistically significant activation cluster only in the left SII cortex. The movement at 9 Hz yielded activations in left and right SII cortex, but also in the left pars triangularis (BA45; MNI-coordinates, center of mass: -46.7, 33.4, 6.2) and near the right insula (BA6, MNI-coordinates, center of mass: 42.3, 1.9, -13.4).

At the individual level in the SI cortex, movements at 0.3 Hz elicited significant activations in 3/11 individuals (voxel-wise, initial threshold: *p* < 0.001, uncorrected; cluster-wise threshold *p* < 0.05, FDR-corrected), at 1 Hz in 7/11 individuals, at 3 Hz in 10/11 individuals, at 6 Hz in 10/11 individuals, at 9 Hz in 10/11 individuals, at 12 Hz in 10/11 individuals. In the SII cortex, movements at 0.3 Hz elicited activations in 3/11 individuals, at 1 Hz in 10/11 individuals, at 3 Hz in 8/11 individuals, at 6 Hz in 7/11 individuals, at 9 Hz in 9/11 individuals and at 12 Hz in 7/11 individuals.

### Response Time-Courses

**FIGURE 4:**
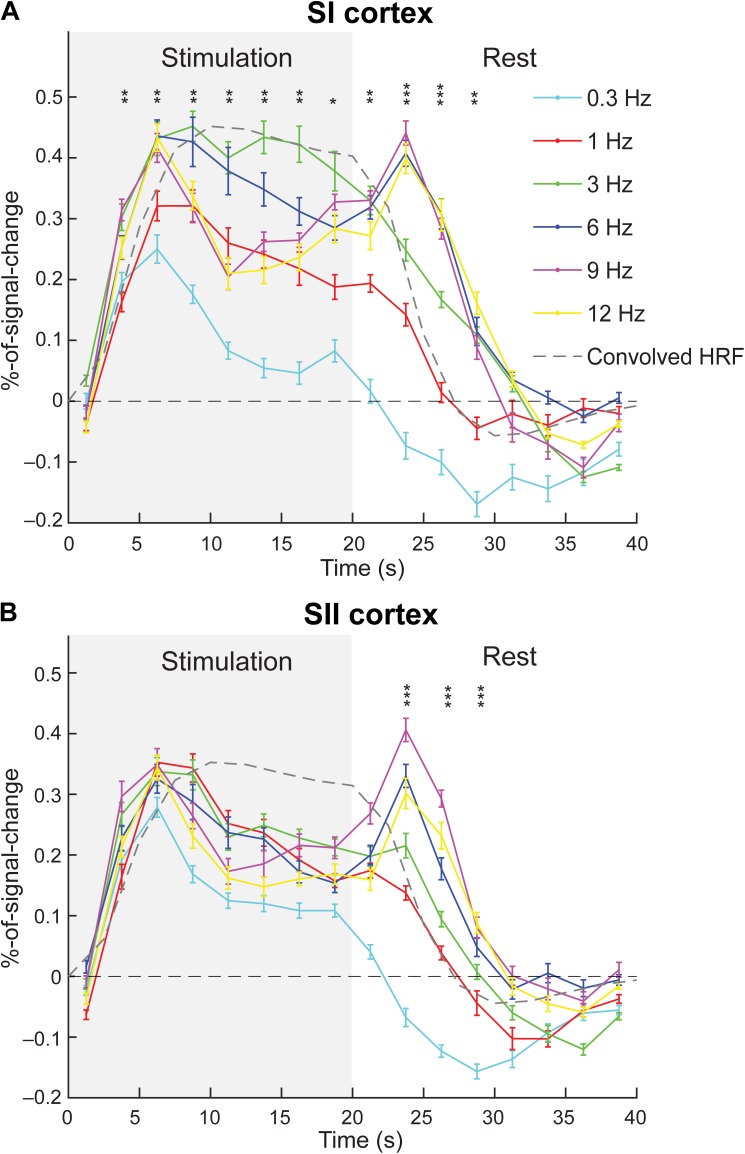
Group-averaged hemodynamic time-courses. The time-course of each movement frequency (solid lines) is shown for SI **(A)** and SII **(B)** cortex. Gray dashed line represents convolved canonical HRF. Gray background represents the stimulation (movement) period. The error bars represent standard errors. The asterisks represent significant differences between the frequencies at a given time points (^∗^*p* < 0.05, ^∗∗^*p* < 0.01, and ^∗∗∗^*p* < 0.001).

Figure 4 shows the group-average hemodynamic time-courses. The first peak of signal change occurred ∼5 s after the onset of the stimulation both in SI and SII cortices for all frequencies. The higher frequencies (6, 9, and 12 Hz) exhibited an offset peak that was evident in both SI and SII cortices ∼5 s after the offset of stimulation.

When comparing the actual time-courses to the convolved cHRF, the actual time courses reached maximum earlier (at 5–7.5 s) than as cHRF (at 10 s). It is not clear, however, whether this difference is significant in reality since the convolved HRF is quite near its peak value at the time point where the actual fMRI-time courses peaked. Moreover, it seems that the convolved HRF continues to plateau and decreases only slightly during the stimulation block. In contrast, the actual time-courses of the movements at lowest (0.3 Hz) and highest frequencies (9 and 12 Hz) seem to decay sharply after their initial peak. Lower to intermediate frequencies (1, 3, and 6 Hz) seem to better follow the convolved HRF. This clear difference was confirmed by statistical comparison of different movement frequencies across the time points. The 3-Hz movements showed higher percent signal change than 9- and 12-Hz movements in time points after the peak response (Figure 4 and Table 2).

**Table 2:** Differences in percentage-of-signal-change between the movement frequencies across time-points.

Time-point (s)																		
Frequency			1.25	3.75	6.25	8.75	11.25	13.75	16.25	18.75	21.25	23.75	26.25	28.75	31.25	33.75	36.25	38.75
**SI**	0.3 Hz																	
		1 Hz	–	1.00	**<0.05**	**<0.01**	**<0.01**	**<0.01**	**<0.001**	0.198	**<0.05**	0.345	0.750	0.460	**–**	**–**	**–**	**–**
		3 Hz	–	**<0.01**	**<0.001**	**<0.001**	**<0.001**	**<0.001**	**<0.001**	**<0.01**	**<0.001**	**<0.001**	**<0.05**	**<0.01**	–	–	–	–
		6 Hz	–	**<0.05**	**<0.001**	**<0.01**	**<0.01**	**<0.001**	**<0.001**	**<0.05**	**<0.001**	**<0.001**	**<0.001**	**<0.05**	–	–	–	–
		9 Hz	–	**<0.001**	**<0.001**	0.085	0.425	**<0.01**	**<0.001**	**<0.01**	**<0.001**	**<0.001**	**<0.001**	**<0.01**	–	–	–	–
		12 Hz	–	**<0.05**	**<0.001**	**<0.01**	0.058	**<0.01**	**<0.001**	**<0.01**	**<0.05**	**<0.001**	**<0.001**	**<0.001**	–	–	–	–
	1 Hz																	
		3 Hz	–	**<0.01**	0.173	**<0.05**	0.111	**<0.01**	0.134	0.063	**<0.05**	**<0.05**	**<0.05**	**<0.05**				
		6 Hz	–	<**0.05**	0.050	0.653	0.865	0.157	0.261	0.240	0.106	**<0.001**	**<0.001**	0.112	–	–	–	–
		9 Hz	–	**<0.001**	0.214	0.295	**<0.05**	0.775	0.919	0.051	0.063	**<0.001**	**<0.001**	**<0.01**	–	–	–	–
		12 Hz	–	**<0.05**	0.173	1.0000	0.359	0.775	0.919	0.163	0.701	**<0.001**	**<0.001**	**<0.01**	–	–	–	–
															–	–	–	–
	3 Hz																	
		6 Hz	–	0.379	0.619	0.110	0.141	0.200	0.740	0.564	0.617	**<0.01**	**<0.05**	0.460				
		9 Hz	–	0.314	0.926	**<0.01**	**<0.01**	**<0.05**	0.147	0.870	0.732	**0.001**	**<0.01**	0.612	–	–	–	–
		12 Hz	–	0.566	1.00	**<0.05**	**<0.05**	**<0.05**	0.147	0.719	0.082	**<0.05**	**<0.05**	0.425	–	–	–	–
	6 Hz														–	–	–	–
		9 Hz	–	**0.05**	0.593	0.140	**<0.05**	0.083	0.292	0.491	0.732	0.570	0.705	0.269				
		12 Hz	–	0.783	0.620	0.653	0.297	0.083	0.292	0.796	0.237	0.677	0.847	0.112				
	9 Hz														–	–	–	–
		12 Hz	–	0.094	0.926	0.295	0.243	1.000	1.000	0.641	0.136	0.345	0.750	0.612	–	–	–	–
**SII**	0.3 Hz																	
		1 Hz	**–**	**–**	**–**	**–**	**–**	**–**	**–**	**–**	**–**	**<0.05**	**<0.01**	0.148				
		3 Hz	–	–	–	–	–	–	–	–	–	**<0.01**	**<0.001**	**<0.01**	–	–	–	–
		6 Hz	–	–	–	–	–	–	–	–	–	**<0.001**	**<0.001**	**<0.01**	–	–	–	–
		9 Hz	–	–	–	–	–	–	–	–	–	**<0.001**	**<0.001**	**<0.01**	–	–	–	–
		12 Hz	–	–	–	–	–	–	–	–	–	**<0.001**	**<0.001**	**<0.001**	–	–	–	–
	1 Hz																	
		3 Hz	–	–	–	–	–	–	–	–	–	0.327	0.331	0.148	–	–	–	–
		6 Hz	–	–	–	–	–	–	–	–	–	**<0.001**	**<0.01**	0.094	–	–	–	–
		9 Hz	–	–	–	–	–	–	–	–	–	**<0.001**	**<0.001**	0.122	–	–	–	–
		12 Hz	–	–	–	–	–	–	–	–	–	**<0.001**	**<0.01**	**<0.05**	–	–	–	–
	3 Hz																	
		6 Hz	–	–	–	–	–	–	–	–	–	**<0.01**	**<0.05**	0.822	–	–	–	–
		9 Hz	–	–	–	–	–	–	–	–	–	**<0.001**	**0.001**	0.870	–	–	–	–
		12 Hz	–	–	–	–	–	–	–	–	–	**<0.01**	**<0.05**	0.431	–	–	–	–
	6 Hz																	
		9 Hz	–	–	–	–	–	–	–	–	–	0.051	**<0.05**	0.870	–	–	–	–
		12 Hz	–	–	–	–	–	–	–	–	–	0.837	0.683	0.600	–	–	–	–
	9 Hz																	
		12 Hz	–	–	–	–	–	–	–	–	–	**<0.05**	0.081	0.513	–	–	–	–

*Non-significant Friedman test results are indicated with a dash. For significant Friedman test results, FDR-corrected Conover’s post hoc tests for individual frequencies results are given. P-values given by the post-hoc-test which are significant, are in bold.*

In the SI cortex, the amplitudes (i.e., percent signal change levels) of different time points differed between movement frequencies at 3.75–28.75 s. (i.e., from 2nd to 12th time point, Figure 4). Table 2 presents *p*-values for comparisons between the frequencies for individual time points. The amplitude at 0.3 Hz frequency was weaker than at most of the other frequencies during the period of 3.75–28.75 s. Similarly, the amplitude at 1 Hz frequency was weaker than all of the higher frequencies at 3.75 s after of the onset of stimulation and during the second peak at 23.75–26.25 s. There were some differences also between the movements at higher frequencies. The amplitude at 3-Hz movement differed from the 9 and 12-Hz movements between 8.75 and 13.75 s.

In SII cortex, there were no statistically significant differences between the amplitudes of the hemodynamic responses at different frequencies during the stimulation period. However, significant differences were detected after the stimulation offset at 23.75 until 28.75 s (Figure 4). For *p*-values from the FDR-corrected *post hoc* tests between the movement frequencies see Table 2.

### The Effect of Stimulation Duration

**FIGURE 5:**
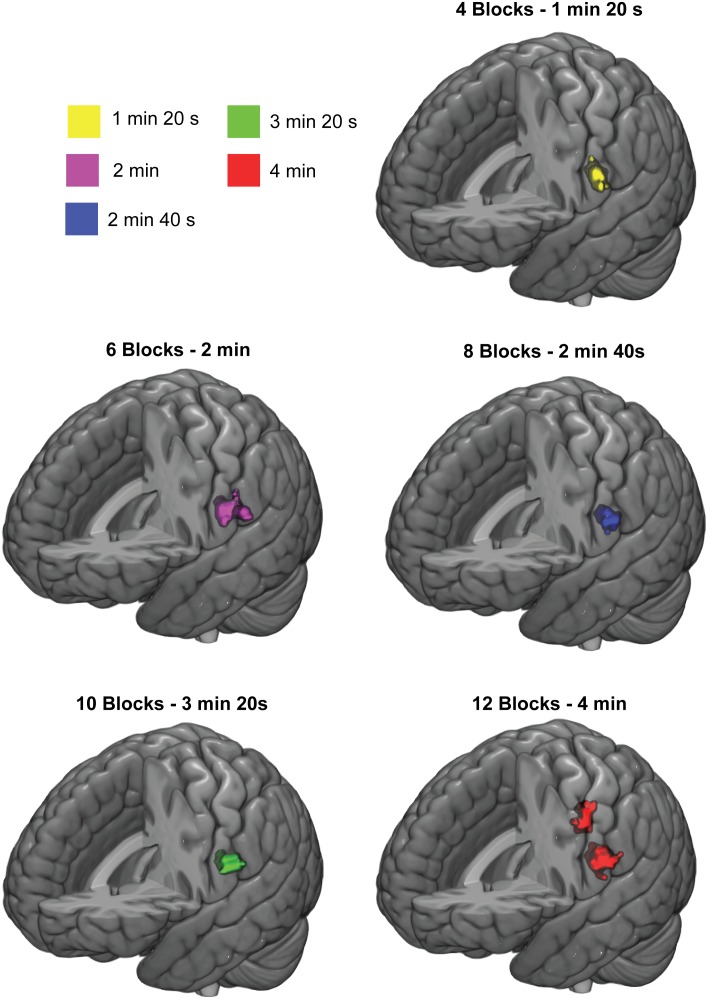
Response clusters for different stimulation durations. Significant group-level activation clusters are colored. Cluster-wise threshold was 0.05 (FDR-corrected) and voxel-wise threshold was 0.001 (uncorrected).

Figure 5 shows the effect of stimulation duration on group-level activation clusters at 3-Hz stimulation. For the SI cortex, only a full stimulation duration of 4 min (12 blocks, total scanning duration 8 min with rest blocks included) was sufficient to elicit a significant activation cluster. For the SII cortex, however, all stimulation durations from 1 min 20 s (4 blocks, total scanning duration 2 min 40 s with rest blocks included) to 4 min (12 blocks) were sufficient to elicit a significant activation cluster in the SII cortex.

## Discussion

Movements at frequencies ≥ 3 Hz were the most efficient to evoke cortical proprioceptive responses in fMRI. The frequency of the finger movements affected the response strength in SI cortex, but not in SII cortex. The initial velocities of the movements were very similar at different frequencies. However, increase in the total movement range enhanced the response strength in SI cortex. As expected, most of the movement frequencies activated SI and SII cortices contralateral to the stimulated finger. Inspection of the hemodynamic-time-course dynamics revealed a secondary response peak after the stimulation offset at ≥ 6 Hz movements. A 4-min stimulation duration was required to elicit significant group-level activation clusters in SI cortex, whereas the shortest tested stimulation duration of 1 min 20 s was sufficient to elicit significant clusters in SII cortex. Lastly, passive movement elicits activations also regions outside SI and SII cortices. We limited our analysis to SI and SII cortices because these are the primary cortices in processing of the proprioceptive afference and we did not observe significant group-level-cluster activations in other cortical regions.

### Movement Frequency Affects Response Strength Only in SI Cortex

In SI cortex, the response strength increased as the movement frequency increased from 0.3 to 3 Hz. However, movement frequencies ≥ 3 Hz elicited activations that were approximately of the same magnitude. In other words, the optimal movement frequency was between 3 and 12 Hz. This range is in-line with earlier fMRI findings for tactile stimulation in humans (Hlushchuk et al., 2015) and rats (Sanganahalli et al., 2008). They found the optimal stimulation frequency to fall between 3 and 4 Hz. Furthermore, these results are in concordance with median-nerve stimulation, in which the response strength is increased when the stimulation frequency was increased from 0.2 to 4 Hz in PET (Ibáñez et al., 1995), from 0.5 to 4 Hz in fMRI (Ferretti et al., 2005) or from 0.2 to 3–5 Hz in fMRI (Iramina et al., 2001). In studies using higher stimulation frequencies, the responses plateaued or even slightly decreased as the stimulation frequency was increased above 3 (Iramina et al., 2001; Manganotti et al., 2009) or 4 Hz (Ibáñez et al., 1995).

When making inferences about the response strength, it should be kept in mind that our measure of response strength is based on mean beta-values, which basically measures how well the BOLD-signal follows the theoretical cHRF. When looking at the level of percent signal change at the first peak (6.25 s) of group-averaged-signals at different frequencies, there are no statistically significant differences between the frequencies > 0.3 Hz (Figure 4 and Table 2). This suggests that at least some of the differences between response strengths might be due to the frequencies ≥ 3 Hz following the theoretical cHRF better than the lower frequencies.

Median nerve stimulation elicits responses in the somatosensory cortices that are arising from a mixture of tactile and proprioceptive afferents. Therefore, it was not clear *a priori* that the median nerve stimulation and proprioceptive stimulation would yield similar correspondence regarding the stimulation frequency. It should be noted, however, that during passive movements some cutaneous tactile afferents are inevitably activated. All in all, it appears that the BOLD-responses of the SI cortex peak around 3-Hz stimulation regardless of somatosensory modality and this relationship might even apply across species (Sanganahalli et al., 2008).

Studies comparing cortical responses recorded with electroencephalography (EEG) and PET using median nerve stimulation (Ibáñez et al., 1995), or MEG and fMRI using electric stimulation of the thumb activating tactile receptors (Iramina et al., 2001) found out that both BOLD and regional cerebral blood flow (rCBF) signals peaked when using stimulation frequencies of 3–4 Hz. This was in contrast to their MEG and EEG results that showed strongest responses at stimulation frequencies of 0.2–2 Hz decreasing after further increases in stimulation frequency. This discrepancy between the MEG/EEG and fMRI/PET is because MEG/EEG measures millisecond scale electrophysiological changes of neural populations whereas fMRI/PET measures several second scale haemodynamics.

Moreover, a neural phenomenon called sensory gating, where the response amplitude is diminished to the second stimulus when two identical stimuli are presented with short intervals in MEG/EEG, might also play a role in the discrepancy between MEG/EEG and fMRI/PET (for a review on sensory gating, see Cromwell et al., 2008). Due to the differences on how the response strength is measured in MEG versus fMRI and the differing time-scales of the BOLD-responses and electrophysiological responses, sensory gating might affect MEG response strength more than fMRI responses leading to longer optimal ISI durations for MEG.

Typically, in MEG or EEG, maximum responses may be elicited only at ISIs of several seconds or even 10s of seconds, depending on the cortical area, and sensory modality (Hari et al., 1982; Lu et al., 1992; Mäkelä et al., 1993; Uusitalo et al., 1996; Wikström et al., 1996). For instance, using passive movements in MEG, Smeds et al. (2017) concluded that the optimal ISI to maximize the signal-to-noise ratio in a fixed measurement time appears to be 1.5–3.0 s (i.e., 0.33–0.67 Hz) for index finger movements.

It has been shown earlier using median nerve stimulation in fMRI that the response strength is dependent on the frequency of the stimulation in SI cortex, but independent of it in the SII cortex (Backes et al., 2000; Ferretti et al., 2005). Our results are in line with these results. One plausible explanation is a difference in the functional roles of SI and SII cortices. In general, SI cortex is more sensitive to variations in stimulus frequency and amplitude than SII cortex (Backes et al., 2000; Nelson et al., 2004; Ferretti et al., 2005; Keysers et al., 2010). Furthermore, it has been suggested based on single-cell-recordings from monkeys that whereas SI cortex processes simple stimulus features only, SII cortex also processes multimodal judgments and more cognitive aspects of sensory processing, such as retaining relevant aspects of the afference in the working memory (Lemus et al., 2010; for a review, see Pleger and Villringer, 2013).

When interpreting fMRI data, one must be careful with this interpretation of SII cortex response being invariant to stimulus frequency since the coding of the movement rate might not be directly related to hemodynamic activation level in the cortex *per se*—but to the intricate interactions of its neuronal populations. For instance, timings or rhythms of spiking-activity might code movement frequency instead of the overall activity level. Moreover, any subdivision of SII cortex sensitive to the movement frequency may be effaced when averaging over the voxels within the ROI.

With the aforementioned reservations in mind, the SII cortex seems indeed to be minimally affected by the stimulation frequency. Thus, in the context of passive movement, it would be important to elucidate what are the stimulus features that SII cortex preferably responds to. Future studies using passive movement to study proprioception could examine a wider variety of stimulus features and tasks including multisensory stimuli and cognitive tasks accompanying the stimuli. For instance, a study where an oddball paradigm is implemented using passive movement might reveal details about the functional role of SII cortex. Using a similar design, Chen et al. (2008) demonstrated that for non-painful and painful somatosensory nerve stimulation, only SII (but not SI cortex) cortex responded to the rarity of stimuli.

### Movement Range Affects Response Strength in SI Cortex

The total movement range was inevitably reduced (from ∼8 to ∼1 mm) with the increase of movement frequency (from 0.3 to 12 Hz). This was because the pneumatic muscle was not able to deflate entirely at the higher frequencies. Nevertheless, the initial velocities of the movements, at different frequencies, were the same. Thus, we did not expect the variation in the movement range to significantly affect the response strength. For example, muscle spindles are activated by only 5-μm change in the length of their parent muscle (Brown et al., 1967). However, we performed a control experiment to confirm whether the movement range affects the response strength or not. Increase of the movement range from 1 to 7 mm enhanced the response strength in SI cortex, but not significantly in SII cortex. In short, the relationship between SII cortex activation and the total movement range was not evident. This is well in line with findings that SII cortex is less sensitive than SI cortex to the low-level stimulus features (Backes et al., 2000; Nelson et al., 2004; Ferretti et al., 2005; Keysers et al., 2010).

### Response Locations Were Mainly in Contralateral SI and SII Cortices

The response locations were mostly in anatomically expected cortical regions following the somatotopy. Somatosensory afferents relay the information from the proprioceptors primarily via the ventral-posterior superior nucleus of the thalamus (Craig, 2008) to areas 3a (BA3a) and 2 (BA2) of the SI cortex. These areas respond to joint movements (Burchfiel and Duffy, 1972; Schwartz et al., 1973) and passive stretching of muscles (Lucier et al., 1975). BA3a is located at the bottom of the central sulcus and BA2 at the anterior bank of the postcentral gyrus. In addition, BA2 receives cortico-cortical inputs both from BA3a (Pons and Kaas, 1986) and Brodman’s areas 1 and 3b, that receive primarily cutaneous afference (Kaas, 1983). The SII cortex is located in parietal operculum and receives input throughout SI cortex and some direct input from somatosensory afferents via the thalamus (Eickhoff et al., 2010).

Our results are primarily in line with previous studies using median nerve (Ferretti et al., 2005) or vibrotactile (Li Hegner et al., 2007) stimuli that indicated significant contralateral SI cortex and bilateral SII cortex responses. In the group-level, an expected significant contralateral SI cortex activation cluster was observed, but only a contralateral SII activation instead of expected bilateral one. This was the case for all ≥3-Hz movements, except for 9-Hz movement that did not reach significant contralateral SI cortex response but had a bilateral SII activation. The lack of significant bilateral SII activation every stimulation frequency except for 9 Hz might be due to insufficient stimulation/scanning time since other studies have reported bilateral SII activation.

The 9-Hz movement elicited responses also in bilateral BA 45. The left BA45 which is also part of the Broca’s area has been implicated in linguistic production and processing (Heim et al., 2003, 2005; Whitney et al., 2009) and semantic tasks (Friederici et al., 2000). The pars triangularis has also been implicated in action observation of finger movements (Molnar-Szakacs et al., 2005) and grasping (Johnson-Frey et al., 2003). A neighboring region within the Broca’s area, the pars opercularis (BA44) has been activated by voluntary finger movements based on visual movement cues (Krams et al., 1998). In their review Binkofski and Buccino (2004) concluded that in addition to its role in language, the Broca’s area might also serve as a high-level sensorimotor integration interface that integrates somatosensory input especially from the hand and face with the ongoing cognitive tasks. As to why the 9-Hz movements activated this region during the passive movements, we do not know. In addition, 9-Hz movement activated also the right insula. Insula has been implicated in wide range of functions including emotional, cognitive and social processing, multimodal integration as well as gustatory, nociceptive, interoceptive, and somatosensory (including proprioceptive) processing (for a review, see Janeš, 2015; for a meta-analysis, see Kurth et al., 2010). Some evidence have been found for human ventral somatosensory area (Eickhoff et al., 2006). Since this lies medially and anterior to the traditional SII cortex, it is possible, this is the region activated by the 9 Hz condition.

### Movement Frequency Affected the Dynamics of the Response Time-Courses

The movements evoked clear signal change in SI and SII cortices. The hemodynamic percent signal change reached first maximum in SI and SII cortices at around 5 s after onset of the stimulation. This corresponds well to the latency of a typical haemodynamic response. After the first peak, the 3-Hz movement stimulus sustained its response amplitude better throughout the stimulation than the movements at other frequencies. This sustained response is in concordance with the results by Iramina et al. (2001) who revealed that 3–5 Hz electric stimulation of the thumb yielded BOLD-responses in the SI cortex that sustained their activation level for significantly longer after stimulation onset than other stimulation frequencies in the 0.2–100 Hz range.

The time-courses showed unexpected offset response for ≥ 6-Hz movements, which may be related to habituation and/or fusing of the proprioceptive perception. It should be noted, however, that the higher frequencies (≥ 6-Hz) also caused the artificial muscle to be partially inflated the whole time during the stimulation and deflate at the end of the stimulation causing a reverse movement back to the resting-position whereas the lower frequencies did not (see Figure 1). This might be the cause of the haemodynamic response at the end of the stimulation instead of an offset response. Still, a similar offset response has been observed for auditory stimulation when using short sound bursts at 35 Hz and higher stimulation frequencies (Harms and Melcher, 2002), i.e., at around stimulation frequencies where participants’ subjective experience of the sound became fused. The neuronal mechanisms for the offset response are not entirely clear. A plausible explanation is that stimulation at the higher frequencies leads to stronger and faster habituation of the neuronal circuits, and they might thus adopt the stimulation as a constant of input, which requires minimal processing in the system. Normal physiological range for voluntary movements is up to ∼6 Hz. It seems therefore reasonable, that the high movement frequencies, which are still achievable voluntarily, would elicit the strongest responses (i.e., 3–12 Hz). It is worth to note, that the movements at 12 Hz are still felt as separate movements.

In SI cortex, some habituation probably occurred at above 6-Hz movements since the hemodynamic signal clearly decayed after the initial peak response (∼5 s after onset of stimulation). When the stimulation ceased, the habituated state was disrupted most likely causing the offset response. This process could also be described in terms of predictive coding, where the cessation of stimulation leads to an increased prediction error that may also produce an offset response (for details see Jehee and Ballard, 2009). However, habituation and predictive coding are likely just different point-of-views to the same neurophysiological phenomenon.

### Benefits, Limitations and Practical Recommendations

The pneumatic-movement actuator has several benefits, but also limitations. It is EEG, MEG, TMS and MRI compatible, durable, provides few millisecond accuracy in timing and stable stimuli. The frequency of the movement is easy to adjust; however, the adjustability of the movement range and velocity are limited, but can be somewhat facilitated with digitally controlled air valves. The pneumatic muscle can compress up to ∼25% of its resting length. The movement range in the current actuator was from 1 mm (at 12 Hz) to 8 mm (at 0.3 and 1 Hz). This limitation is caused by the artificial muscle not having enough time to deflate entirely at the higher frequencies thereby limiting the effective movement range. Fortunately, the proprioceptors, sensing the movement in our musculoskeletal system are extremely sensitive. For example, during vibration, muscle spindles are activated by only 5-μm change in the length of their parent muscle (Brown et al., 1967).

When adjusting movement frequency of continuous movements, it is inevitable that either the movement range or velocity is modified. In our case, it was the movement range that was modified. The movement range varied from ∼1 mm (at 12 Hz) to ∼8 mm (at 0.3 Hz). In our control experiment, we found that increase in the total movement range enhanced the response strength in SI cortex, but not in SII cortex. It appears that the movement frequency, rather than range, is more important when optimizing the response strength in fMRI. The 0.3-Hz and 1-Hz movements had by far the largest movement ranges (>8 mm), but the weakest responses in SI cortex. Moreover, the 12-Hz movement with only ∼1 mm movement range elicited similar response strength in SI cortex as the 3-Hz movement with ∼5 mm movement range. In our control experiment, 5 mm and 7 mm movements at fixed 2-Hz movement elicited the strongest responses in the SI cortex. However, it is possible that our approach underestimates the response strengths of 6, 9, and 12 Hz since at these frequencies the movement ranges were the lowest. Finally, we cannot rule out possible interaction effects of the movement frequency and range since the movement frequency was kept constant at 2 Hz in our control experiment.

A limitation of this study was a small sample size of 11 participants. Thus, statistical power and generalizability is somewhat limited. It is likely that we have not detected all subtle effects of the movement parameters on cortical proprioceptive processing, for example activations in the other relevant cortical regions. Thus, our focus was on SI and SII cortices. It should be noted that passive movements activate other sensorimotor cortices such as primary motor cortex (M1; Xiang et al., 1997; Druschky et al., 2003; Terumitsu et al., 2009; Piitulainen et al., 2015) and supplementary motor area (SMA; Weiller et al., 1996). These regions were not included in our ROI analysis.

It is also noteworthy that passive movements do activate the proprioceptors in muscles, tendons and around the joints, but do not necessarily activate entirely the same cortical networks as during active voluntary movements or during kinesthetic perception. For instance, joint position judgment (Bevan et al., 1994), shape discrimination, i.e., haptics (for a review, see Sathian, 2016), or mirror-matching of position of one’s arm to the other persons arm (see Kenzie et al., 2017) all utilize varying components of proprioception or sensorimotor integration in a way passive movement does not. Our movement actuator can only be used to study the component of passive movement in proprioception.

Lastly, SI cortex required longer duration of total stimulation than SII cortex to obtain significant activation clusters on the group-level. In fact, anything less than the longest stimulation duration used (4 min, 12 blocks) was insufficient to elicit significant activation cluster in SI cortex. For SII cortex, the shortest stimulation duration (1 min 20 s, 4 blocks) was sufficient to elicit significant an activation cluster in the left hemisphere. Thus, the sufficient stimulation varies between the cortical regions. It should be noted, however, that the sufficient stimulation duration might depend on additional factors such as block length or whether the movement frequency is constant or varied randomly. It is reasonable to assume that neuronal processes related to the habituation effects play a role also in the sufficient stimulation time. Finally, it should be noted that when using 20 s rest block like we did, the total scanning duration is at least double of the total stimulation duration (i.e., the sufficient scanning duration is at least 8 min for SI cortex and at least SII 2 min 40 s for SII cortex with the block structure we used).

## Conclusion

Movements ≥ 3 Hz elicited the strongest responses in SI cortex, with no frequency dependency in SII cortex during proprioceptive stimulation. In addition, increase in the total movement range enhanced the response strength in SI cortex, but did not have significant effect in SII cortex. The movement frequency appears to have a stronger effect on the fMRI response strength than the total range of the passive movement. We recommend frequencies between 3 and 6 Hz and movement range of ∼5 mm or higher for future studies. Total stimulation duration per condition should be at least 4 min when obtaining group-level activation clusters from SI cortex. For SII cortex, total stimulation duration of 1–2 min might be sufficient for passive movement of the finger. The higher movement frequencies (≥6 Hz) likely introduced a habituation effect, demonstrated with a clear stimulus-offset response in the time-courses of the hemodynamic responses. Finally, our study demonstrates the feasibility of using the pneumatic-movement actuators for the hand in fMRI to examine of the proprioceptive processing in the human brain.

## Author Contributions

TN, HP, and LH designed the experimental design. TN and HP piloted the fMRI design. TN and HP did the fMRI recordings. TN analyzed the data. TN, LH, and HP wrote the manuscript.

## Conflict of Interest Statement

The authors declare that the research was conducted in the absence of any commercial or financial relationships that could be construed as a potential conflict of interest.

## Abbreviations

(f)MRI: (functional) magnetic resonance imaging
GTO: golgi tendon organ
ISI: inter-stimulus-interval
MEG: magnetoencephalography
PET: positron emission tomography
SI cortex: primary somatosensory cortex
SII cortex: secondary somatosensory cortex
